# Diagnostic performance of 3D SPACE for comprehensive knee joint assessment at 3 T

**DOI:** 10.1007/s13244-012-0197-5

**Published:** 2012-10-26

**Authors:** Pieter Van Dyck, Jan L. Gielen, Filip M. Vanhoenacker, Eline De Smet, Kristien Wouters, Lieven Dossche, Paul M. Parizel

**Affiliations:** 1 Department of Radiology, University Hospital Antwerp, Wilrijkstraat 10, 2650 Edegem, Belgium; 2 Department of Radiology, University of Ghent, De Pintelaan 185, 9000 Ghent, Belgium; 3 Department of Radiology, AZ St-Maarten Duffel/Mechelen, Rooienberg 25, 2570 Duffel, Belgium; 4 Department of Biostatistics, University Hospital Antwerp, Wilrijkstraat 10, 2650 Edegem, Belgium; 5 Department of Orthopedics, University Hospital Antwerp, Wilrijkstraat 10, 2650 Edegem, Belgium

**Keywords:** SPACE, Knee, 3 T, Cartilage, Meniscus

## Abstract

**Objective:**

To assess the diagnostic performance of 3D sampling perfection with application-optimised contrasts using variable flip-angle evolution (SPACE) turbo spin-echo (TSE) sequences compared to 2D TSE for comprehensive knee assessment at 3 T.

**Methods:**

From January to July 2011, isotropic 3D SPACE was added to a 2D knee protocol at 3 T. Forty patients underwent subsequent arthroscopy. Three readers independently assessed MR images for meniscus, anterior cruciate ligament (ACL) and cartilage lesions. Readers 1 and 2 evaluated 3D and 2D data at separate sittings; reader 3 interpreted the complete exam including 3D and 2D sequences. Accuracies were calculated using arthroscopy as reference standard. McNemar’s test (*p* < 0.05) was used to compare 3D and 2D techniques.

**Results:**

The highest diagnostic yield was obtained by reader 3 (accuracies ≥88 %). For the medial meniscus, readers performed better with the 2D technique than with 3D SPACE (accuracies 85–88 % vs. 78–80 %, respectively) (*p* > 0.05). For the lateral meniscus and ACL, 3D and 2D techniques had similar performance (accuracies ≥93 %). For cartilage lesions, 3D SPACE had significantly lower specificity (*p* = 0.0156) than the 2D protocol for one reader.

**Conclusion:**

The conventional 2D TSE acquisition is more reliable than 3D SPACE for comprehensive assessment of the knee at 3.0 T.

***Main Messages*:**

• *3D SPACE is a valuable component of a knee MR protocol at 3 T.*

• *3D SPACE cannot be used as a single sequence in the MR evaluation of the knee at 3 T*.

• *Knee MR protocols at 3 T should include both 2D and 3D TSE sequences*.

## Introduction

Three-dimensional (3D) turbo spin-echo (TSE) sequences with isotropic resolution have recently been developed and are now commercially available on many magnetic resonance (MR) vendor platforms [1]. These sequences include 3D fast spin-echo (FSE) Cube (GE Healthcare), 3D Fourier Transform (FT, Philips Medical Systems) and sampling perfection with application-optimised contrasts using different flip-angle evolutions (SPACE, Siemens Medical Systems) [1–3]. The advantage of the new 3D TSE acquisitions is their capability of mimicking the contrast properties of conventional two-dimensional (2D) TSE proton-density weighted acquisitions [4]. In addition, high-quality multiplanar reformatted (MPR) images may be created in any orientation from the volumetric source data. However, uncertainty remains as to whether a single 3D TSE acquisition has potential for replacing the multiple conventional 2D acquisitions currently used. Although the first clinical results on the diagnostic performance of 3D isotropic resolution TSE sequences were encouraging [3, 5], the most recently published studies have described potential limitations of these sequences for evaluating the knee joint [6, 7]. Thus, additional studies are needed to determine the diagnostic usefulness of 3D TSE in future knee MR protocols at 3 T. The purpose of this retrospective study was to assess the diagnostic performance of the 3D TSE SPACE sequence, as compared to routine 2D TSE sequences, for evaluating the menisci, anterior cruciate ligament (ACL) and cartilage of the knee joint in symptomatic patients at 3 T.

## Materials and methods

### Patient selection and medical record review

This retrospective study correlating MR imaging with arthroscopy findings was performed with a waiver of informed consent from the institutional review board. All MR examinations of the knee performed at our institution with a single 3-T MR system from January to July 2011 were reviewed. Patients included in our study met the following criteria: (1) they had undergone a 3-T MR of the knee consisting of 2D TSE sequences and the 3D SPACE sequence; (2) they had an available medical record with the relevant clinical history; (3) they had no prior history of knee surgery; (4) they had subsequent knee arthroscopy. After elimination of patients on the basis of these criteria, we identified a group of 40 patients (25 male, 15 female; average age 43 years, range 18-78 years) eligible for this study. The mean time interval between MR and arthroscopy was 46 days (range 3–112 days).

### MR imaging protocol

All MR knee examinations were performed with a single 3-T system (Trio TIM Magnetom; Siemens Healthcare, Erlangen, Germany) and an eight-channel phased-array knee coil with the same imaging protocol. The imaging protocol consisted of standard 2D TSE acquisitions and a SPACE 3D TSE acquisition with the imaging parameters of all sequences summarised in Table 1. The 2D protocol consisted of a coronal fat-suppressed (FS) TSE intermediate-weighted (IM-w) acquisition, an axial FS TSE IM-w acquisition and a coronal SE T1-weighted acquisition. The 3D protocol consisted of a single 3D TSE acquisition in the sagittal plane with the commercially available SPACE sequence. The SPACE isotropic source data were post-processed on a high-performance workstation (Leonardo, Siemens Healthcare) to create sagittal, coronal and axial MPR images with 1-mm slice thickness. Moreover, readers were free to use volumetric data to create MPRs in any orientation and slice thickness.

**Table 1:** Parameters for MR imaging sequences

Parameter	3D TSE	2D TSE		
	Sagittal	Coronal	Axial	Coronal
Repetition time (ms)	1200	3560	3670	450
Time to echo (ms)	47	22	23	9.8
Matrix size	320 × 320	250 × 384	307 × 384	279 × 448
Field of view (mm)	180	180	160	160
Slice thickness (mm)	0.65	3	3	3
Interslice gap (mm)	–	0.3	0.3	0.3
Bandwidth (Hz/pixel)	391	191	191	191
Echo- train lenght	46	7	7	2
Fat suppression	SPAIR	FS	FS	–
Signal averages	1	1	1	1
Acceleration factor	2	2	2	2
Imaging time (min:s)	10:51	1:07	1:24	1:41

*TSE* turbo spin-echo, *SPAIR* spectral adiabatic inversion recovery, *FS* spectral fat suppression

### MR image analysis

Three radiologists who had between 10 and 25 years of experience in musculoskeletal radiology and who were blinded to clinical and arthroscopic results at the time of review retrospectively and independently assessed MR images for meniscus, ACL and cartilage lesions. Readers 1 and 2 evaluated 3D and 2D data sets at separate sittings with a 4-week interval to minimise recall bias. During the first review, they used the SPACE sequence with MPR images to detect meniscal, ACL and cartilage lesions within the knee joint. During the second review, the readers used the 2D sequences to detect these joint abnormalities. Reader 3 interpreted the complete MR exam including the 3D and 2D sequences at one sitting. The diagnostic criterion for meniscal tear was abnormal signal intensity within the meniscus that definitely extended to the meniscal articular surface on one or more sections or abnormal morphology of the meniscus [8]. If a meniscal tear was diagnosed on MR, the observers localised tears in the anterior horn, body or posterior horn of the meniscus in order to make sure that the tear identified at MR imaging was the same as that identified at arthroscopy. ACL tears were diagnosed on MR imaging on the basis of the presence of increased signal intensity in the ligament. If ligament margins were intact, the tear was termed partial. If fibre disruption could clearly be detected in the anteromedial (AM) or posterolateral bundle (PL) of the ACL, an isolated bundle tear was reported [9]. If margins were not identified or there was ligament retraction and no identifiable central ligament was present, the tear was termed complete [10]. Cartilage abnormalities were graded using the Noyes classification system [11]. Only cartilage lesions grade 3 and 4 were recorded for the purpose of this study. Six cartilage compartments (medial femoral, medial tibial, lateral femoral, lateral tibial, patellar and femoral trochlea) were assessed separately.

### Arthroscopic knee surgery

All arthroscopic procedures were performed at our institution by one of three experienced orthopaedic surgeons who specialised in sports medicine and knee surgery and who had between 10 and 25 years of clinical experience. Standard anteromedial and anterolateral portals were used with blunt probing of both menisci and the ACL to evaluate their integrity. Once identified, the location of a meniscal tear was recorded (anterior horn, body and/or posterior horn). The ACL was classified as either normal, partially torn or completely torn. If possible, partial discontinuity of ACL fibres was located in the AM or PL bundle [9]. All articular surfaces of the knee joint were graded at arthroscopy by using the Noyes classification system [11]. The orthopaedic surgeons were aware of the prospective interpretations of the MR imaging studies in all patients at the time of arthroscopy.

### Statistical analysis

For each reader and each imaging series, the sensitivity, specificity and accuracy of MR, with corresponding 95 % confidence intervals, were calculated using arthroscopy as the standard of reference. McNemar’s test was used to identify differences between 3D and 2D TSE sequences for the diagnosis of meniscal and ACL tears as well as cartilage lesions. Differences were considered to be significant if the *p*-value was less than 0.05. For the assessment of interobserver agreement, kappa (к) coefficients were calculated. According to the recommendations of Landis and Koch [12], к -values were interpreted as slight (к = 0.0–0.20), fair (к = 0.21–0.40), moderate (к = 0.41–0.60), substantial (к = 0.61–0.80) or excellent (к = 0.81–1.0). All analyses were performed using the Statistical Package for Social Sciences for Windows (version 17.0; SPSS Inc., Chicago, IL, USA).

## Results

**Fig. 1:**
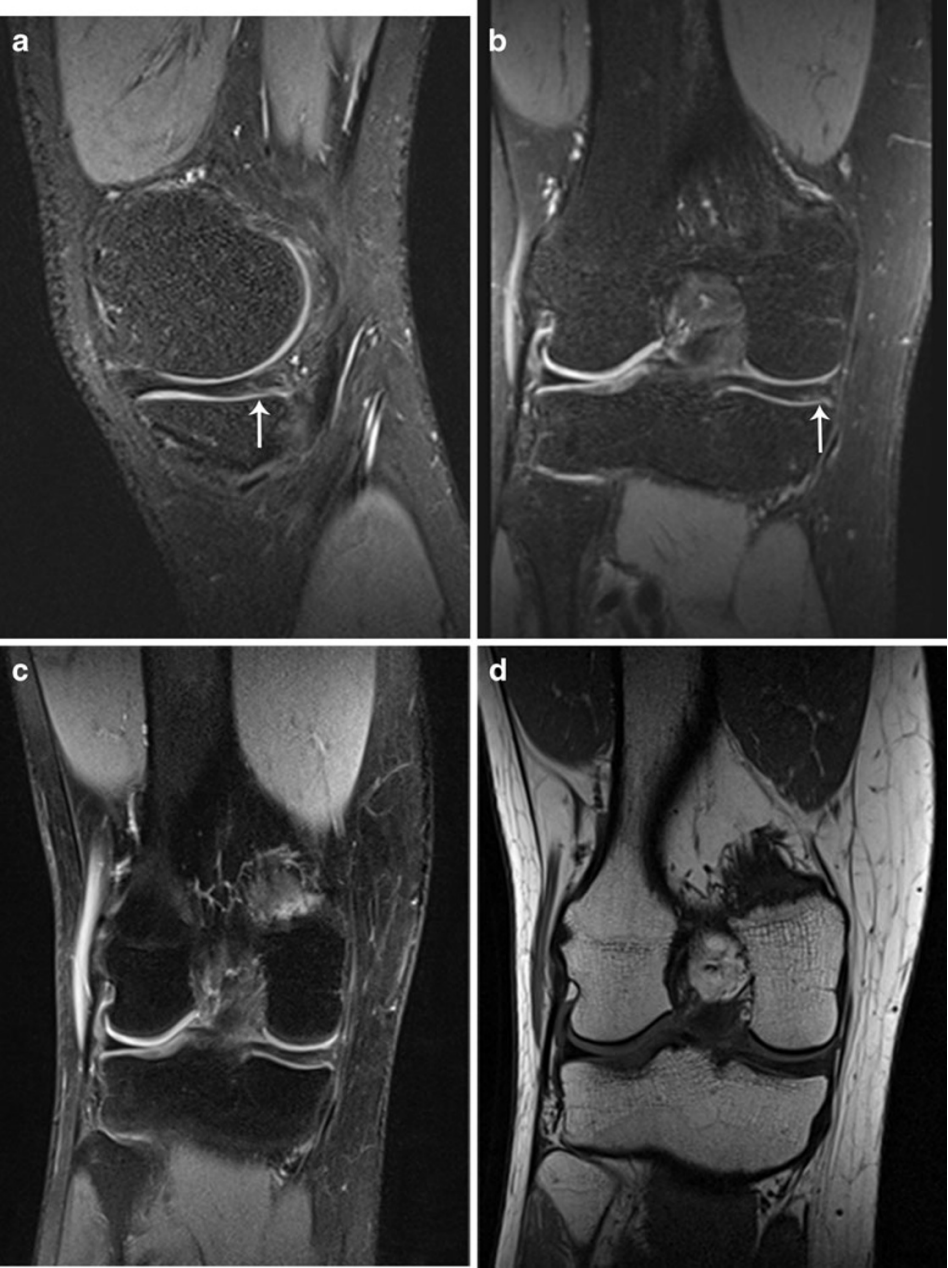
A 21-year-old male with a surgically confirmed normal medial meniscus, which was interpreted as a meniscal tear by all readers using 3D SPACE and as a normal meniscus by all readers using the routine MR protocol. Sagittal (**a**) and coronal (**b**) 3D SPACE images demonstrate grade 3 signal in the posterior horn of the medial meniscus extending to the inferior surface (*arrow*). Coronal 2D TSE intermediate-weighted FS (**c**) and T1-weighted (**d**) images show normal medial meniscus. Poor image contrast on 3D SPACE images was considered the primary cause of the discrepancy

**Fig. 2:**
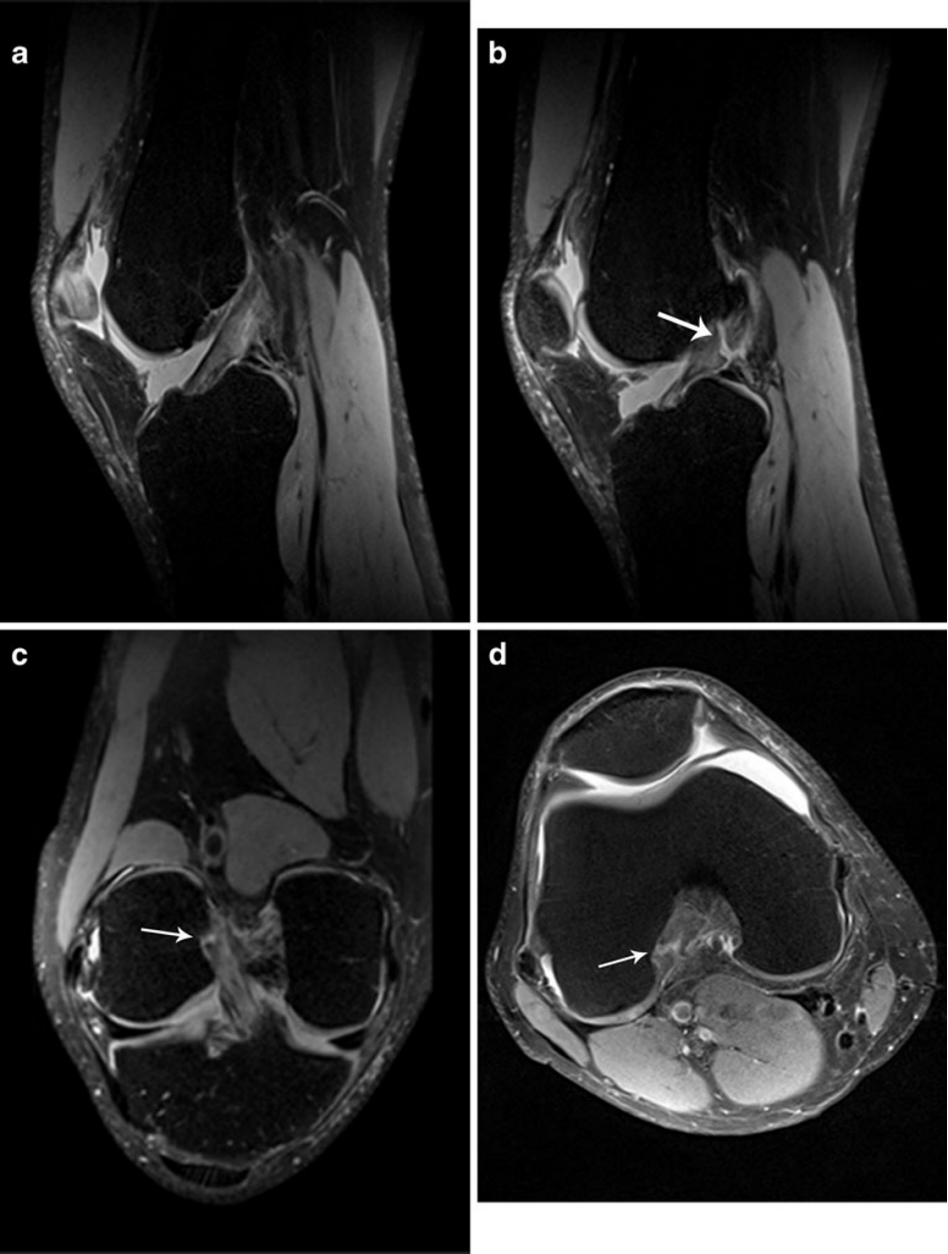
A 53-year-old male with a surgically confirmed tear of the posterolateral (PL) bundle of the ACL, which was correctly identified by all readers using both 3D SPACE and the routine MR protocol. Parasagittal (**a**–**b**) and coronal (**c**) 3D SPACE images show intact anteromedial and torn PL (*arrow*) bundle of the ACL. Axial 2D TSE intermediate-weighted FS (**d**) image also demonstrates tear of the PL bundle of the ACL (*arrow*)

**Fig. 3:**
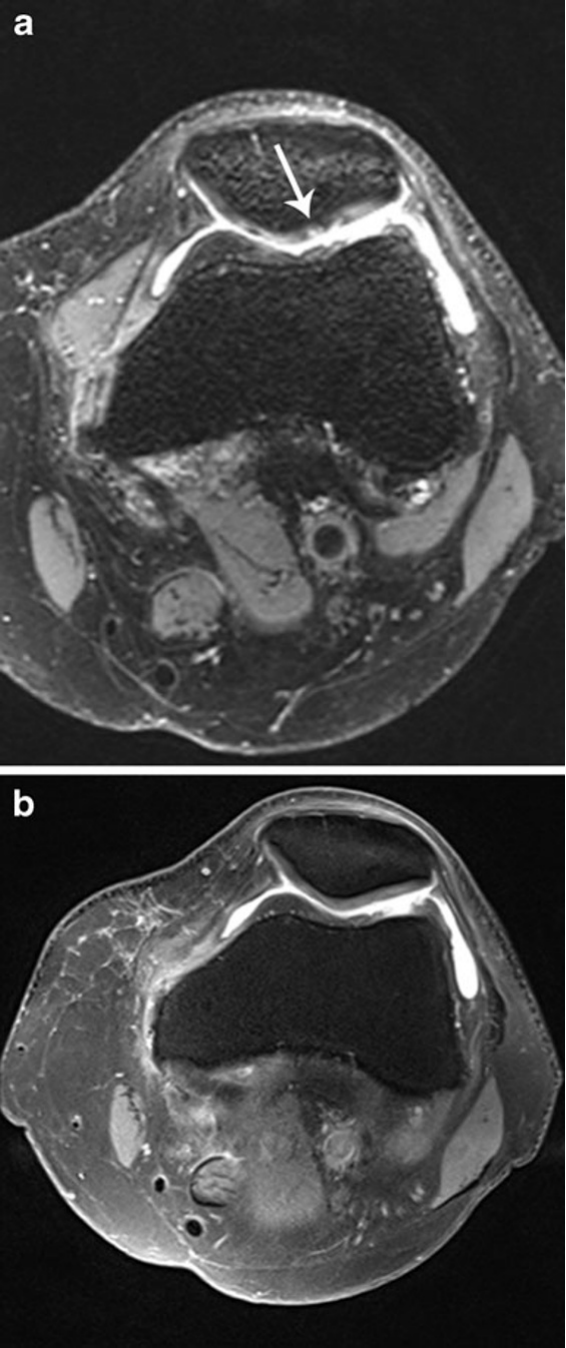
A 50-year-old female with surgically confirmed normal patellar cartilage, which was interpreted as grade 3 cartilage lesions by all readers using 3D SPACE and as normal patellar cartilage by all readers using the routine MR protocol. Axial 3D SPACE (**a**) shows an irregular patellar articular surface (*arrow*). Axial 2D TSE intermediate-weighted FS (**b**) image shows a smooth patellar articular surface. Poor image contrast on 3D SPACE images was considered the primary cause of the discrepancy

Arthroscopy revealed 24 tears of the medial meniscus, 8 tears of the lateral meniscus, 10 ACL (9 complete and 1 isolated PL bundle) tears and 21 grade 3–4 cartilage lesions (medial tibia, *n* = 1; medial femur, *n* = 5; lateral tibia, *n* = 3; lateral femur, *n* = 2; patella, *n* = 7; trochlea, *n* = 3). Tables 2 and 3 show the sensitivities, specificities and accuracies, with corresponding 95 % confidence intervals, of the 3D SPACE and 2D TSE sequences for the MR diagnoses rendered by the three readers. The highest diagnostic yield was obtained by reader 3 (accuracies ≥ 88 % for all lesions). For the medial meniscus, both readers 1 and 2 performed better with routine 2D acquisition than with 3D SPACE acquisition (accuracies 85–88 % and 78–80 %, respectively). This difference was not statistically significant. There were five false-positive MR interpretations of medial meniscal tear for both reader 1 and 2 using 3D SPACE (specificity 69 %) (Fig. 1). In the detection of eight lateral meniscal tears, the 3D and 2D techniques had similar performance (accuracy 95 %). Both reader 1 and 2 missed one tear in the posterior horn of the lateral meniscus using only 2D sequences. In the detection of ten ACL tears, readers 1 and 2 had similar performance with the 3D and 2D techniques (accuracies 93–100 %). One partial (PL bundle) ACL tear was correctly identified by all readers using both 3D and 2D sequences (Fig. 2). There were three false-positive MR interpretations of (partial) ACL tear for reader 1 using 2D sequences. For detecting cartilage lesions within the knee joint, reader 1 had similar performances with the 3D and 2D acquisition methods. However, we found significantly lower specificity (*p* = 0.0156) for reader 2 using 3D SPACE compared to 2D sequences for evaluating the patellofemoral compartment [90 % (63/70) and 100 % (70/70), respectively] (Fig. 3). Both the 3D and 2D sequences had excellent interobserver agreement for meniscus and ACL lesions (к > 0.81), and moderate interobserver agreement for cartilage lesions (к = 0.60).

**Table 2:** Diagnostic performance of 3D SPACE and routine MR imaging protocol in the detection of meniscus and anterior cruciate ligament lesions for all readers

	Reader 1			Reader 2			Reader 3
	2D	3D	*p*-value	2D	3D	*p*-value	Complete exam
Medial meniscus							
Sensitivity	83 [64–93] (20/24)	88 [69–96] (21/24)	1,000	88 [69–96] (21/24)	83 [64–93] (20/24)	1,000	92 [74–98] (22/24)
Specificity	94 [72–99] (15/16)	69 [44–86] (11/16)	0.125	81 [57–93] (13/16)	69 [44–86] (11/16)	0.500	81 [57–93] (13/16)
Accuracy	88 [74–95] (35/40)	80 [64–90] (32/40)	0.375	85 [71–93] (34/40)	78 [63–88] (31/40)	0.250	88 [74–95] (35/40)
Lateral meniscus							
Sensitivity	88 [53–98] (7/8)	100 [68–100] (8/8)	1,000	88 [53–98] (7/8)	100 [68–1] (8/8)	1,000	100 [68–100] (8/8)
Specificity	97 [84–99] (31/32)	94 [80–98] (30/32)	1,000	97 [84–99] (31/32)	94 [80–98] (30/32)	1,000	94 [80–98] (30/32)
Accuracy	95 [84–99] (38/40)	95 [84–99] (38/40)	1,000	95 [84–99] (38/40)	95 [84–99] (38/40)	1,000	95 [84–93] (38/40)
Anterior Cruciate							
Sensitivity	100 [72–100] (10/10)	100 [72–100] (10/10)	1,000	100 [72–100] (10/10)	100 [72–100] (10/10)	1,000	100 [72–100] (10/10)
Specificity	90 [74–97] (27/30)	100 [89–100] (30/30)	0.250	100 [89–100] (30/30)	100 [89–100] (30/30)	1,000	100 [89–100] (30/30)
Accuracy	93 [80–97] (37/40)	100 [91–100] (40/40)	0.250	100 [91–100] (40/40)	100 [91–100] (40/40)	1,000	100 [91–100] (40/40)

Sensitivity, specificity and accuracy data are percentages with number/total in parentheses. Data in brackets are lower and upper bounds of 95 % confidence intervals. *P*-value < 0.05 indicates a significant difference

**Table 3:** Diagnostic performance of 3D SPACE and routine MR imaging protocol in the detection of cartilage lesions for all readers

	Reader 1			Reader 2			Reader 3
	2D	3D	*p*-value	2D	3D	*p*-value	Complete exam
Overall cartilage							
Sensitivity	71 [48–89] (15/21)	76 [53–92] (16/21)	1,000	81 [58–95] (17/21)	81 [58–95] (17/21)	1,000	81 [58–95] (17/21)
Specificity	94 [90–96] (205/219)	95 [91–97] (208/219)	0.6072	99 [96–100] 216/219	95 [92–98] (209/219)	0.0923	99 [96–100] (216/219)
Accuracy	92 [87–95] (220/240)	93 [89–96] (224/240)	0.4807	97 [94–99] (233/240)	94 [90–97] (226/240)	0.1185	97 [94–99] (233/240)
Tibiofemoral compartment							
Sensitivity	64 [31–89] (7/11)	64 [31–89] (7/11)	1,000	73 [39–94] (8/11)	73 [39–94] (8/11)	1,000	73 [43–90] (8/11)
Specificity	95 [91–98] (142/149)	97 [92–99] (144/149)	0.7539	98 [94–100] (146/149)	98 [94–100] (146/149)	1,000	98 [94–99] (146/149)
Accuracy	93 [88–97] (149/160)	94 [90–97] (151/160)	0.7744	96 [92–100] (154/160)	96 [92–99] (154/160)	1,000	96 [92–98] (154/160)
Patellofemoral compartment							
Sensitivity	80 [44–97] (8/10)	90 [55–100] (9/10)	1,000	90 [55–100] (9/10)	90 [55–100] (9/10)	1,000	90 [60–98] (9/10)
Specificity	90 [80–96] (63/70)	91 [82–97] (64/70)	1,000	100 [92–100] (70/70)	90 [80–96] (63/70)	0.0156*	100 [95–100] (70/70)
Accuracy	89 [80–95] (71/80)	91 [83–96] (73/80)	0.6875	99 [93–100] (79/80)	90 [81–96] (72/80)	0.0156*	99 [93–100] (79/80)

Sensitivity, specificity and accuracy data are percentages with number/total in parentheses. Data in brackets are lower and upper bounds of 95 % confidence intervals

*P-value < 0.05 indicates a significant difference

## Discussion

Current knee MR protocols typically consist of 2D TSE sequences repeated in multiple planes. These sequences have excellent tissue contrast and high in-plane spatial resolution [1]. However, they have relatively thick slices and small gaps between slices that can obscure pathology secondary to partial volume averaging [1, 2]. Three-dimensional sequences can reduce partial volume averaging by acquiring thin, continuous slices through joints [2, 4]. Most 3D sequences with isotropic resolution described in the literature were gradient-echo sequences because of its short TR. However, gradient-echo imaging has major drawbacks, including accentuated magnetic susceptibility and lack of contrast between abnormal and normal tissue [1, 2, 13, 14]. Recently, 3D TSE sequences have become available allowing for comprehensive knee joint assessment because of its high tissue contrast and slice resolution [1]. These acquisitions, typically used at 3 T, entail a variable flip angle refocussing pulse and allow extremely large turbo factors [2, 4]. Few studies have directly compared 3D and 2D TSE sequences for comprehensive knee joint assessment [3, 5, 7, 13]. Therefore, we undertook this study to determine the diagnostic value of 3D SPACE as compared to routine 2D TSE sequences for the assessment of internal derangements of the knee joint at 3 T.

In our study, readers performed better with conventional 2D acquisition than with 3D SPACE acquisition for evaluation of the menisci of the knee with 3.0-T MR imaging. Although the differences did not reach statistical significance, we found that a conventional 2D TSE protocol was more accurate than an isotropic 3D SPACE protocol for the evaluation of the medial meniscus. The false-positive MR interpretations of medial meniscal tear (*n* = 5 for both readers) using the 3D SPACE protocol were related to poor image contrast and blurring on SPACE images. Our study results are in concordance with the findings of Kijowski et al. [6] and Subhas et al. [7] who also found the 2D TSE technique to be more accurate in the evaluation of the knee meniscus as compared to 3D TSE. Also, Ristow et al. [15] found a decreased visualisation of low contrast structures such as bone marrow and menisci because of a higher amount of image blurring and indistinctness of the structural edges on 3D TSE images.

The 3D SPACE sequence may have advantages over 2D sequences for evaluating the knee ligaments. The thin, continuous sections of 3D SPACE minimise the effect of partial-volume averaging, which can be a source of diagnostic error when evaluating the ACL of the knee [3]. However, in our study, 3D SPACE had similar sensitivity, specificity and accuracy as the routine MR imaging protocol in the detection of nine complete and one partial ACL tear.

Our study findings are in line with prior studies demonstrating high diagnostic accuracy of 3D SPACE for evaluating the articular cartilage of the knee joint (overall accuracies ≥93 %) [3, 13, 16, 17]. However, 3D SPACE had significantly lower specificity (*p* = 0.0156) than the routine MR protocol for detecting patellofemoral cartilage lesions for one radiologist (reader 2). This lower specificity of 3D SPACE is most likely related to decreased in-plane spatial resolution and image blurring due to acquisition of high spatial frequencies late in the echo train. This may cause a normal articular surface to appear indistinct and ill defined, simulating the appearance of cartilage degeneration [13].

Recently, in a study by Notohamiprodjo et al. [18], the SPACE sequence was further optimised and used in combination with a 15-channel knee coil for 3D imaging of the knee at 3 T. These authors found a considerable refinement of image quality of this optimised 3D SPACE sequence with increased in-plane resolution and reduction of image blurring as compared to the non-optimised version of 3D SPACE with the eight-channel coil.

Our study had several limitations. First, an important limitation was the small patient population. However, chance fluctuations causing differences in MR accuracy can occur even with sample sizes as large as 100 [19]. In this era of limited resources and cost savings in health care, a study including more than 100 patients would not be possible in our busy clinical practice. We believe that, without reaching statistical significance, our study found clinically significant results demonstrating that 3D SPACE is a good but not superior sequence compared to currently used 2D sequences for detecting cartilage and ACL lesions and that 3D SPACE is less accurate compared to routine MR for detecting meniscal lesions. Second, we did not obtain 2D sagittal images because of time constraints. However, accurate assessment of the knee joint could be made using coronal and axial 2D sequences, as indicated by the high accuracy rates obtained by reader 1 and 2 for all lesions. Moreover, replacing the sagittal 2D sequence by the sagittally oriented 3D SPACE sequence did not decrease the accuracy of our knee MR protocol at 3 T. This is evidenced by the accuracy rates obtained by reader 3, all being well within the range of previously reported accuracy rates at 3 T. Third, we compared sequences with different slice thicknesses and spatial resolutions. However, we did not want to reconstruct the 3D SPACE images with a larger slice thickness as we wanted to assess the full potential of the small slice thickness. Moreover, in an earlier study by Notohamiprodjo et al. [3], it was found that SPACE 1-mm MPRs were superior to SPACE 2-mm MPRs for visualisation of anatomic structures. Fourth, we only assessed grade 3 and grade 4 cartilage lesions according to the Noyes classification system. However, low-grade cartilage lesions are diagnosed with less accuracy during arthroscopy, making it an imperfect gold standard for identification of these lesions [20]. Fifth, the arthroscopy findings could have been biased by the availability of the MR reports introducing surgical bias and limiting the reference standard. Sixth, selection bias was introduced, as our study group consisted of only a proportion of all patients undergoing MR of the knee at our institution.

Given our results, we believe the present study adds to the increasing pool of data suggesting that 3D SPACE may be a valuable component of a knee MR protocol at 3 T. However, the 3D SPACE sequence needs further optimisation regarding image quality and further acceleration of acquisition for improving time efficiency and patient comfort. Until this goal is achieved, 3D SPACE cannot be used as a single sequence in the MR evaluation of the knee at 3 T.

In conclusion, conventional 2D acquisition is more reliable compared to 3D SPACE for comprehensive assessment of the knee joint at 3.0 T.
